# Efficient production of human bivalent and trivalent anti-MUC1 Fab-scFv antibodies in *Pichia pastoris*

**DOI:** 10.1186/1472-6750-9-70

**Published:** 2009-08-11

**Authors:** Steve Schoonooghe, Vladimir Kaigorodov, Monika Zawisza, Caroline Dumolyn, Jurgen Haustraete, Johan Grooten, Nico Mertens

**Affiliations:** 1 Department for Molecular Biomedical Research, VIB, Technologie Park 927, B-9052 Ghent, Belgium; 2 Molecular Immunology Lab, Ghent University, Technologie Park 927, B-9052 Ghent, Belgium

## Abstract

**Background:**

Tumour associated antigens on the surface of tumour cells, such as MUC1, are being used as specific antibody targets for immunotherapy of human malignancies. In order to address the poor penetration of full sized monoclonal antibodies in tumours, intermediate sized antibodies are being developed. The cost-effective and efficient production of these molecules is however crucial for their further success as anti-cancer therapeutics. The methylotropic *P. pastoris *yeast grows in cheap mineral media and is known for its short process times and the efficient production of recombinant antibody fragments like scFvs, bivalent scFvs and Fabs.

**Results:**

Based on the anti-MUC1 PH1 Fab, we have developed bivalent PH1 bibodies and trivalent PH1 tribodies of intermediate molecular mass by adding PH1 scFvs to the C-terminus of the Fab chains using flexible peptide linkers. These recombinant antibody derivatives were efficiently expressed in both mammalian and *P. pastoris *cells. Stable production in NS0 cells produced 130.5 mg pure bibody and 27 mg pure tribody per litre. This high yield is achieved as a result of the high overall purification efficiency of 77%. Expression and purification of PH1 bibodies and tribodies from *Pichia *supernatant yielded predominantly correctly heterodimerised products, free of light chain homodimers. The yeast-produced bi- and tribodies retained the same specific activity as their mammalian-produced counterparts. Additionally, the yields of 36.8 mg pure bibody and 12 mg pure tribody per litre supernatant make the production of these molecules in *Pichia *more efficient than most other previously described trispecific or trivalent molecules produced in *E. coli*.

**Conclusion:**

Bi- and tribody molecules are efficiently produced in *P. pastoris*. Furthermore, the yeast produced molecules retain the same specific affinity for their antigen. These results establish the value of *P. pastoris *as an efficient alternative expression system for the production of recombinant multivalent Fab-scFv antibody derivatives.

## Background

Monoclonal antibodies (MoAbs) targeted at tumour associated antigens (TAAs) are a promising new therapeutic option for treating cancer patients. MoAbs such as Rituximab [1] and Trastuzumab [2] are already becoming part of the standard treatment regimen, targeting TAA^+ ^tumours specifically and with fewer side effects than chemotherapeutics. However, a number of problems that diminish antibody efficacy in a therapeutic setting still need to be addressed. The large size of a full sized antibody slows vascular diffusion and prevents its penetration deep into solid tumours[3,4]. Moreover, radionuclide or cytotoxin coupled molecules persist longer in the general circulation and can thus cause more toxic side-effects [5]. An equally important, yet sometimes overlooked issue is the production of sufficient quantities of MoAb. MoAb therapies involve high doses, usually more than 1 g per patient per year, and can only be generated in relatively expensive mammalian cell fermentors[6,7].

In order to address the poor tumour penetration of full-sized antibodies, MoAb derivatives, such as Fab and scFv fragments, have been generated that, due to their smaller size, penetrate more readily in tissues and can deliver a rapid peak dose at the tumour site[8]. However, molecules smaller than 60 kDa are generally cleared too rapidly from the body to allow sufficient tumour accumulation. Intermediate sized recombinant antibodies, such as the 80 kDa minibodies, still demonstrate good tissue penetration, while not being cleared as rapidly from the blood[9-12]. Most of these molecules lack an Fc effector domain but can be used as blocking agents for growth factor receptors, inducers of apoptosis or as carriers of radiotherapeutic isotopes, toxins, cytokines or other biologically active proteins[13-15]. Several ways of making intermediate sized antibody derivatives have already been described which mainly involve the usage of different dimerisation motifs such as leucine zippers or Fc chain interactions[16-19]. As with MoAbs, obtaining sufficient correctly folded or heterodimerised product for large-scale application often is a bottleneck for these recombinant antibody derivatives. Major efforts are therefore being made in the development of cheap and efficient heterologous expression systems. Over the last few years, yeasts, like *Pichia pastoris*, have gained a significant interest for the production of recombinant antibody fragments using cheap mineral defined media and requiring shorter process times as compared to mammalian cell culture[20,21]. The possibility to grow yeasts to high cell densities of up to 100 g/l dry biomass, along with the availability of strong, inducible promoters, such as the alcohol oxidase gene (AOX1) promoter, are further advantages of heterologous expression in *P. pastoris *[22].

We previously described a novel model to engineer bi- or trivalent antibody derivatives (bi- or tribodies) of intermediate size (75–100 kDa), based on fusion of single-chain variable fragments (scFv) to the C-terminus of one or each of the Fd and L chains of a Fab fragment[23]. Fab-scFv BsAb are efficiently produced in mammalian cells, with 90% of the product being in the correctly heterodimerised form. In this study, we describe the production and purification of the human PH1 Fab-scFv bivalent bibody and Fab-(scFv)_2 _trivalent tribody directed against the MUC1 TAA. This tumour antigen is an under-glycosilated form of MUC1, and is associated with poor prognosis upon overexpression in breast and pancreas cancer[24,25]. Using the Fab-sFv template, we formatted the αMUC1 bi- and tribodies for expression in *P. pastoris*, and compared the *P. pastoris *production system to transient expression in human HEK293T cells and stable expression in mouse NS0 cells. The binding functionalities of both mammalian and yeast derived preparations are gauged demonstrating the functional equivalence of the respective preparations. Combined, these results demonstrate *P. pastoris *to be a viable alternative expression system for Fab-scFv molecules.

## Results

### Construction of PH1 bibody and tribody expression vectors

**Figure 1:**
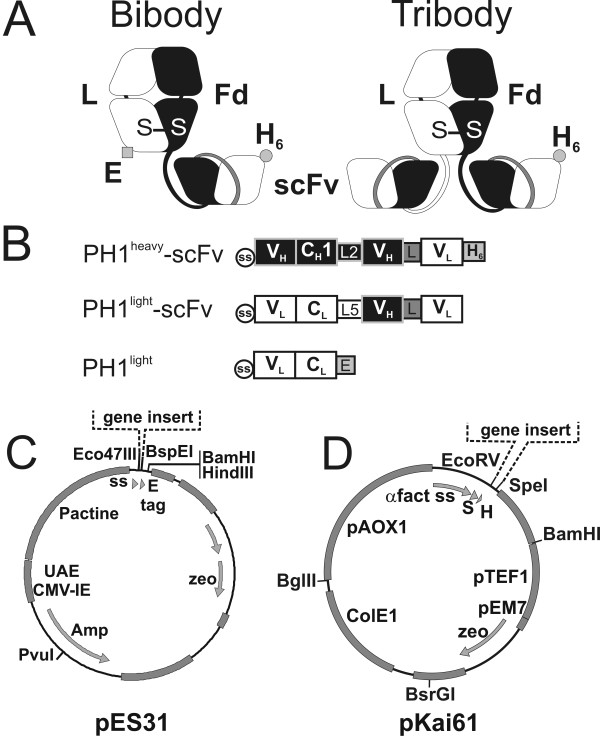
**Characteristics of the PH1 bi- and tribody production systems**. **A**, Schematic presentation of the assembled bibody and tribody molecules. Heavy chains are depicted in black, light chains in white, S-S: sulphur bond, E: E-tag (black square), H_6_: His-tag (black circle). **B**, Construction design for the heavy and light chain bi- and tribody expression cassettes. V_H_: variable heavy chain domain, C_H_1: constant heavy chain domain 1 (black). V_L_: variable light chain domain, C_L_: constant light chain domain (white). ss: signal sequence from BCL-1 (circle), L2: EPSGP(G_4_S)_3 _linker, L5: DVDGGSRGDGPG linker, L: (G_4_S)_3 _linker, E: E-tag (GAPVPYPDPLEPR) and H6: His_6_-tag. **C**, Schematic presentation of the pES31 mammalian expression plasmid carrying the strong βactin-βglobulin promotor. **D**, Schematic presentation of the pKai61 *P. pastoris *expression plasmid. Of note are the methanol induced AOX promoter and the S-tag (S) which is added to the gene insert. Also indicated (dotted line) is the place for gene insertion in the respective expression plasmids (**C, D**).

The bivalent bibody and trivalent tribody targeting the MUC1-TAA were designed using the Fab-sFv model[23]. A bivalent bibody was generated by fusing a PH1 scFv fragment to the C-terminus of the PH1 Fab Fd chain (Figure 1A). For the trivalent tribody, an additional PH1 scFv was fused to PH1 L chain. The combination of PH1^heavy^-scFv and PH1^light ^chains (Figure 1B) leads to the formation of a 78 kDa bibody. Combining the PH1^heavy^-scFv and PH1^light^-scFv chains results in a 104 kDa tribody.

The pES31 vector was constructed to facilitate cloning and expression of bi- and tribody genes in mammalian cells (Figure 1C). To this end, the βactin-βglobulin promoter and CMV enhancer from pCAGGS were introduced in the pCDNA3- plasmid carrying the zeocin resistance gene. To facilitate expression and excretion, a kozak sequence and a consensus excretion signal sequence were introduced in front of the expression cassette. Eco47III, BspEI and BamHI restriction sites were introduced to allow modular exchange of antibody expression cassettes. The PH1^heavy^-scFv and PH1^light^-scFv expression cassettes were subsequently cloned into pES31 for expression in HEK293T and NS0 mammalian host cells.

For the expression of PH1 bi-and tribodies in *P. pastoris*, a single vector based on pKai51 was constructed, with a bicistronic expression cassette for both the light and heavy chain (Figure 1D). pKai51 originated from pGAPZalfaA in which several additional features were introduced, including an AOX1 promotor from pPICZA. The PH1 bibody and tribody light and heavy chains were first introduced into separate pKai51 derived vectors, after which the expression modules for the heavy and light chain were combined in one vector, resulting in the pKai61-BiBody-PH1 and pKai61-TriBody-PH1 vectors.

### Expression of PH1 bibody and tribody in mammalian cells

**Figure 2:**
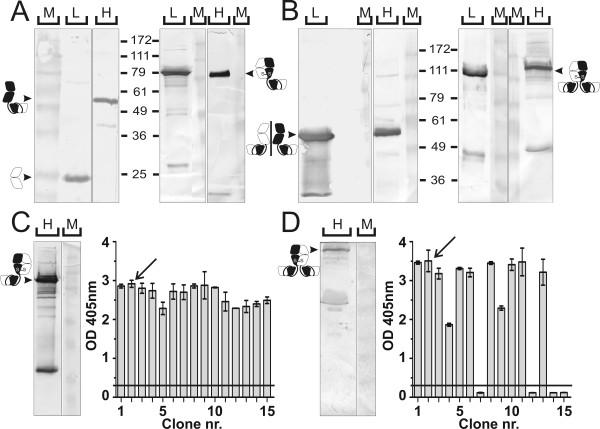
**Expression analysis of PH1 bi- and tribody produced in mammalian cells**. **A, B**, Western blot analyses of ± 1 μg HEK293T produced bibody (**A**) and tribody (**B**) run on a 10% SDS-PAGE gel. Gels to the left of the molecular mass ruler show β-mercaptoethanol reduced samples, to the right unreduced samples are shown. The bands corresponding to the respective antibody products are indicated by arrows and the corresponding antibody chain symbols. L: κ light chain detection, H: His heavy chain detection, M: relative molecular mass marker. **C, D**, Shown on the left is an anti-His western blot analysis of ± 20 μl NS0 produced bibody from clone NS0-Bi2 (**C**) or tribody from clone NS0-Tri2 (**D**) supernatant. Arrows and antibody chain symbols indicate the running height for the respective PH1 derivatives. Shown to the right of the gels are ELISA screenings of culture fluid from 15 NS0-bibody (**C**) or NS0-tribody (**D**) clones on coated mGroEL-MUC1, detected with anti-κ/anti-mouse IgG1-AP and readout at 405 nm. Arrows show the selected clones, the horizontal black lines indicate 3× background value.

The bibody and tribody expression vectors were expressed in the mammalian cell lines HEK293T and NS0. The expression and heterodimeric nature of both molecules was verified through western blot detection of reduced and non-reduced samples with human κ and anti-His specific antibodies. Transient expression of the bibody encoding plasmids in HEK293T cells revealed the expected 52 and 26 kDa molecular mass bands from the PH1^heavy^-scFv and PH1^light ^chains under reducing conditions and the 78 kDa bibody under non-reducing conditions (Figure 2A). Similarly, transient HEK293T expression of the tribody plasmids resulted in a 52 kDa band from the PH1^heavy^-scFv and PH1^light^-scFv chains under reducing conditions and the 104 kDa tribody under non-reducing conditions (Figure 2B). The same bibody and tribody products were obtained following stable expression of the PH1^heavy^-scFv/PH1^light ^and PH1^heavy^-scFv/PH1^light^-scFv plasmid combinations in NS0 cells. Figure 2C shows the western blot detection with His-tag specific antibodies of a non-reduced sample of the PH1 bibody clone NS0-Bi2. Figure 2D shows a similar blot for the PH1 tribody clone NS0-Tri2. Both clones were selected on the basis of ELISA with mGroEL-MUC1 coated plates and anti-human κ detection (Figure 2C, D). Providing a more constant long-term product supply, the stable NS0 transfectants were further used as source for mammalian produced bi- and tribody.

### Purification of mammalian cell produced PH1 bibody and tribody

**Figure 3:**
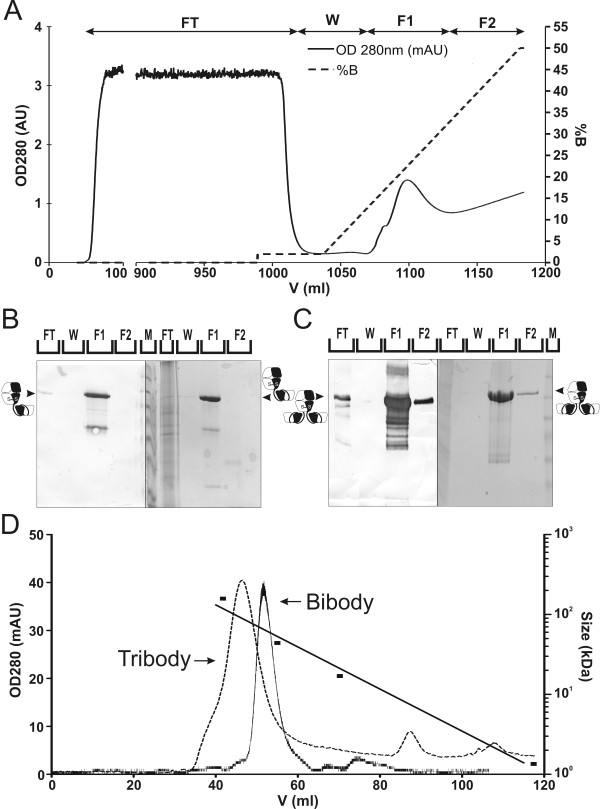
**Purification of NS0 produced PH1 bi- and tribody through IMAC and SEC**. **A**, UV 280 nm (solid line) chromatogram of the serum free NS0 produced PH1 tribody, purified on a 20 ml C16/20 Chelating Sepharose Fast Flow column in 20 mM Phosphate buffer and eluted through an imidazole gradient (dotted line). **B, C**, 10% SDS-PAGE analysis of PH1 bibody (**B**) or tribody (**C**) IMAC fractions from NS0 culture supernatant. The left parts of the gel figures show anti-His MoAb developed blots with 1/1000 of a fraction loaded/lane. On the right, Coomassie stained gels are shown with 1/500 of a fraction/lane loaded. FT: flow through, W: Wash, F1, 2: elution fractions containing a mean of 200 and 400 mM imidazole respectively, M: relative molecular mass marker. The majority of the PH1 products are present in the 200 mM imidazole elution fraction. **D**, SEC purification of bi- and tribody IMAC elution fractions. UV 280 nm chromatogram of the NS0 produced PH1 bibody (solid line) and tribody (dotted line) purified on a C16/60 Sephacryl S-200 column. Arrows depict PH1 bibody and tribody peaks. Also indicated is the Bio-rad molecular mass standard run under similar conditions on the same column (black squares).

PH1-derived bibody and tribody were purified using immobilised metal affinity chromatography (IMAC) for the main purification step. Figure 3A illustrates the fractionation of NS0 produced PH1 tribody onto an equilibrated IMAC column. Western blotting after elution revealed the presence of PH1 bibody and tribody in the 200 mM imidazole fractions (Figure 3B, C). To obtain a higher grade product, we performed SEC to remove any remaining dimers or aggregates. To this end, 2 ml of the eluted fraction of the bi- or tribody was loaded on a Sephacryl S-200 column equilibrated with PBS (Figure 3D). For the bibody, the product was present exclusively as monomers. In the tribody preparation, less than 4% of the original sample was observed as dimers. An estimated 90% of the product loaded on the column could be recovered with an estimated purity of 95%.

Based on colorimetric concentration determination and on the analysis of the Coomassie stained SDS-PAGE gels, the overall efficiency for stably produced PH1 bi- and tribody was estimated at 77% and yielded 130.5 mg bibody and 27 mg tribody per litre NS0 supernatant, starting from 2 l cultures.

### Stable production of PH1 bibody and tribody in P. pastoris

**Figure 4:**
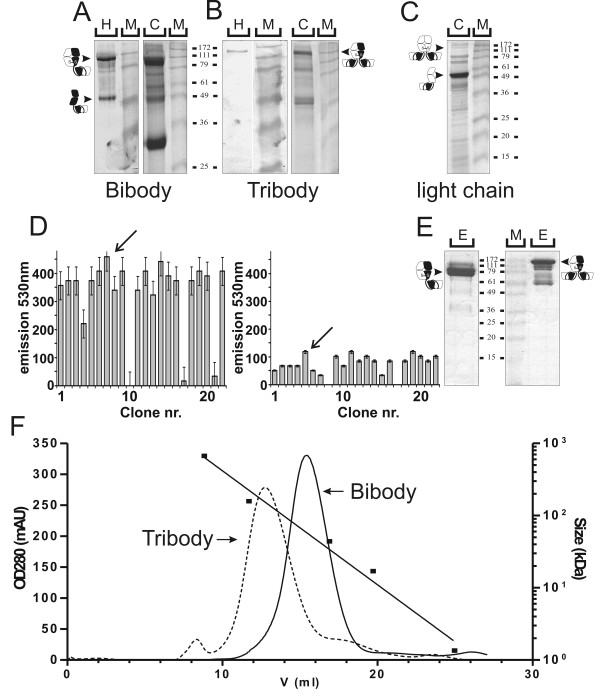
**Characterisation and purification of yeast produced PH1 bi- and tribody**. **A, B**, 12.5% SDS-PAGE characterisation of culture supernatant from the PH1 bibody (**A**) or tribody (**B**) expressing *P. pastoris *cells. Left panels show S-protein-AP detected western blots of 100 μl samples. Right panels show Coomassie detection of 200 μl samples. **C**, Coomassie stained SDS-PAGE gel of 200 μl culture supernatant from PH1-scFv PH1 light chain expressing *P. pastoris *cells. Arrows and antibody chain symbols indicate the running height for PH1 derived proteins. M: relative molecular mass marker. **D**, S-tag assay for expression level screening of 22 *Pichia pastoris *bi- and tribody clones. Left graph shows results for the bibody, right graph for the tribody. Based on these results, bibody clone Pichia-Bi7 and tribody clone Pichia-Tri5 were selected for production. **E**, Coomassie stained 15% SDS-PAGE gel showing 1/500 samples from the IMAC elution fractions of PH1 bibody (left) and tribody (right) purified from ammonium precipitated *Pichia *supernatant. **F**, SEC purification of yeast produced PH1 bi- and tribody. UV 280 nm chromatogram of the *P. pastoris *produced PH1 bibody (solid line) and tribody (dotted line) purified on a Superdex 200 HR10/30 column. Arrows depict PH1 bibody and tribody peaks. Also indicated is the Bio-rad molecular mass standard run under similar conditions on the same column (black squares).

The bicistronic pKai61-BiBody-PH1 and pKai61-TriBody-PH1 vectors were linearised and transformed to *P. pastoris *through electroporation. Westernblot detection with S-protein AP and Coomassie staining of SDS-PAGE gels loaded with unreduced *Pichia *supernatant samples revealed the successful expression of both the PH1 bibody (Figure 4A) and tribody (Figure 4B). As in mammalian cells, no significant amounts of separate heavy chains were observed during bi-and tribody expression. Expression of the PH1^light^-scFv light chain vector alone resulted in production of 52 kDa free light chain monomers and did not result in extensive light chain homodimerisation (Figure 4C). Individual clones were screened by means of an S-Tag assay (Figure 4D). This resulted in the selection for further production of the bibody clone Pichia-Bi7 and tribody clone Pichia-Tri5 which expressed the highest levels of bibody and tribody.

A small scale 6 day production of the bi- and tribody was performed in YPNM with antifoam and 1% methanol added every 12 h. The resulting supernatant was purified using IMAC (Figure 4E), preceded by ammonium sulphate precipitation to remove peptides and other interfering substances from the yeast medium. Further purification over a gelfiltration column of the antibody containing eluate resulted in an estimated 95% pure preparation free of light chain homodimers (Figure 4F), with a total estimated product recovery of 22%. In total 36.8 mg pure bibody per litre of supernatant was recovered from a 250 ml shake-flask culture. For the tribody 12 mg pure product per litre supernatant was recovered from an initial culture volume of 500 ml.

### Binding characteristics of mammalian and yeast cell produced bi- and tribodies

**Figure 5:**
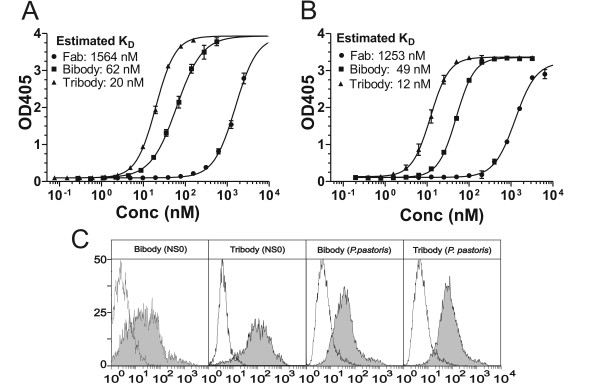
**Binding characteristics of PH1 derivatives. A, B**, ELISA on coated mGroEL-MUC1 peptide. Increasing concentrations of PH1 Fab (circles), bibody (squares) and tribody (triangles) purified from NS0 (**A**) or *P. pastoris *cell cultures (**B**) were allowed to interact with coated mGroEL-MUC1. After 405 nm OD readout, saturation binding curves and constants were calculated using non-linear regression. Shown is the mean ± SD of triplicates. **C**, Binding of PH1 derivatives to cell associated MUC1. Histograms of flow cytometric analysis showing the interaction with MUC1^+ ^OVCAR3 cells of the PH1 bibody and tribody from NS0 or *P. pastoris *origin. Cells were stained with 20 μg/ml PH1 bibody or tribody, followed by mouse anti-human κ (filled) or anti-*c-myc *IgG_1 _(open) isotype control MoAb. Bound MoAbs were detected with Alexa 488-conjugated anti-mouse IgG.

The MUC1 antigen binding capacity and apparent affinity of PH1 bi- and tribodies, produced in NS0 and *Pichia pastoris *cells, were verified by ELISA using recombinant MUC1 peptide as antigen. The monovalent PH1 Fab was used as a reference. As shown in figure 5A, the mammalian produced PH1 bibody demonstrated a 25-fold increase in apparent affinity when compared to the PH1 Fab. This increment was comparable to the values observed for the yeast produced bibody (Figure 5B), thus resulting in K_D _values of approximately 62 nM and 49 nM for the mammalian and yeast bibody respectively. The extra PH1 scFv attached to the PH1 tribody resulted in a further 3-fold increase in apparent affinity in relation to the bibody. Again, this was the case for both mammalian (K_D _≈ 20 nM; Figure 5A) and yeast cell produced (K_D _≈ 12 nM; Figure 5B) tribody products. To further confirm the equivalent binding of the NS0 and *Pichia *produced PH1 bi- and tribody, binding to the MUC1^+ ^tumour cell-line OVCAR3 was examined by flow cytometry. Both PH1 bibody and tribody demonstrated significant binding (Figure 5C) and no apparent differences could be observed between the mammalian and yeast expressed products. Also, no significant binding was detected when MUC1^- ^Lovo cells were used (not shown). These results demonstrate that both production methods yielded products with comparable specific activities.

## Discussion

Alternatively glycosilated MUC1 is overexpressed on a large number of carcinomas and is associated with poor prognosis, making it an interesting target for antibody immunotherapy[26-28]. The human PH1 Fab targeted against the MUC1 TAA was used to develop a human bivalent bibody and trivalent tribody by respectively fusing one or two PH1 scFvs to the C-terminus of the Fab chains. Both molecules are of an intermediate weight, which should slow clearing from the body compared to Fab and scFv fragments, and should also improve tumour penetration compared to MoAbs. As with previously described mammalian expressed bi- and tribodies[23], the HEK293T and NS0 produced PH1 bi- and tribody were expressed almost exclusively as correctly heterodimerised Fab-scFv products, a characteristic attributed to processing in the endoplasmatic reticulum of the L and Fd chains by the mammalian immunoglobulin heavy chain binding protein (BiP) chaperone [29]. However, also expression in *P. pastoris *resulted in the majority of bi- and tribody products being correctly heterodimerised. SDS-PAGE analysis of *Pichia *supernatant revealed, as in mammalian cells[23], the absence of heavy chains released from the cells. This suggests that the yeast Karp2, a Hsp70-class (78 kDa) heat shock protein and analogue of mammalian BiP, plays the same chaperone role as BiP in the endoplasmatic reticulum[30,31], retaining the heavy chains until correctly paired with a light chain. Transfection of the light chain resulted in secretion of mainly L-chain monomers with only a minor fraction of dimers, indicating that the affinity between light chains is limited. By removing the His_6_-tag sequence from the light chain DNA, L-chain homodimers were further excluded during IMAC purification.

In mammalian cells, the use of the strong βactin-βglobulin hybrid promoter and upstream CMV enhancer in pES31 results in increased expression compared to CMV and EF1 promotors [32]. Transient expression of the PH1 bi- and tribody in the pES31 system produced 15.4 mg bibody and 5.77 mg tribody per litre HEK293T medium (not shown). These values are akin to what we described before for mouse bispecific bibodies [32]. The three-fold difference in expression levels between the PH1 bibody and tribody was also observed in the stably transfected mammalian NS0 and yeast cells. Besides the 35% larger size of the tribody, the more complex structure of the molecule and the presence of 3 copies of both the PH1 V_H _and V_L _could hamper the correct cellular expression.

Stable production in NS0 cells clearly resulted in increased production compared to transient expression in HEK293T. In addition, the use of protein free medium raised the overall purification efficacy from 56% for HEK293T productions to 77% for NS0 productions. The peak cell concentration in NS0 batch culture was around 3.10^9 ^cells/l. This leaves the possibility to further increase production by switching to a fed-batch system in which culture conditions are more finely tuned [33]. Although culture media costs have been significantly reduced in the past years, producing antibodies and their derivatives in mammalian cells still is a relatively expensive procedure [34]. Hence, there is a profound interest in alternative production systems based on more primitive cells with shorter process times and the ability to grow to very high cell densities on cheap media. The commonly used prokaryotic E. coli expression systems do not seem suited for the production of the large heterodimeric bi- and tribody molecules. Bibody production in *E. coli *cells resulted in a limited expression levels of 50–250 μg product/l[35]. *Pichia pastoris *has however gained significant interest over the last few years for the production of recombinant antibodies and antibody fragments. This methylotropic yeast can secrete large amounts of recombinant antibody fragments like scFvs, bivalent scFvs and Fabs[20,21,36-38]. The non-human glycosilation patterns of *P. pastoris *are however a problem for the production of complete MoAbs, yet are not an issue for the non-glycosilated bi- and tribodies. Building on this, we successfully produced in *P. pastoris *PH1 bi -and tribodies in viable quantities. Gelfiltration of the *Pichia *products demonstrated that very few dimers and nearly no degradation products were present after IMAC purification. Yet, also in *P. pastoris*, tribody clones were 3 times less productive as compared to bibody clones. However, the surprisingly good production level in *Pichia *of a trivalent antibody with 12 mg pure product/l supernatant may be an advantage when compared to other trispecific or trivalent intermediate sized antibody models, like trispecific single chains[39,40] and triabodies[41,42] that produce in the 0.5 mg/l range in *E. coli *fermentations. Although the yields obtained in *Pichia *medium were comparable to NS0 yields, the low recovery rate from *Pichia *medium of 22% leaves ample room for optimisation of the purification procedures. Replacing the standard ammonium precipitation step, wasting about 50% of the product, with a column based capture step may be a straightforward way to achieve such an increase in recovery. Combined with increases in yield through growing the *Pichia *clones in closely monitored fermentation conditions, further increases in overall production yield are likely to be achieved.

## Conclusion

Independent of the expression system used, the bi- or tribody molecules bound to their MUC1 targets with equal effectiveness either in a peptide context or on OVCAR3 cancer cells, indicating the mammalian and yeast expression products were of similar specific activity.

Combined with the efficient expression and recovery of the yeast produced bi- and tribodies, our results identify *P. pastoris *as a valuable alternative to mammalian expression for the production of recombinant antibody derivatives based on the Fab heterodimerisation platform.

## Methods

### Anti-MUC1 antibody and antigen

The human PH1 Fab[43] and the derived PH1 scFv, both directed against the MUC1 epitope PAPGS were a generous gift from H. Hoogenboom (Dyax, Maastricht, NL). The MUC1 PDTRPAPGS peptide was fused to mGroEL. This GroEL-MUC1 protein is efficiently produced in *E. coli *and purified with IMAC via an N-terminal His_6 _tag (data not shown).

### Cells

HEK293T, a human embryonic kidney cell line transfected with the SV40 large T-Ag (SV40T^tsA1609^)[44], was used for transient eukaryotic expression. NS0 is a mouse myeloma commonly used for hybridoma production or stable recombinant MoAb expression. OVCAR3 cells are derived from a human adenocarcinoma and express high levels of MUC1-TAA. HEK293T and NS0 were grown in DMEM medium containing 10% foetal bovine serum (FBS). Stable NS0 clones were grown in chemically defined hybridoma medium supplemented with synthechol NS0 supplement (Sigma Aldrich, St. Lois, MO). OVCAR3 was cultured in RPMI1640 medium supplemented with 10% FBS and 0.01 mg/ml bovine insulin. Lovo is a human colorectal adenocarcinoma cell-line with low MUC1 expression cultured in Ham's F12K with 10% FBS. The methylotrophic yeast *P. pastoris *strain GS115(his4) was obtained from Invitrogen (Merelbeke, BE), maintained on YPD plates at 4°C and grown in liquid YPD at 30°C.

### Expression plasmids and gene assembly

The pES31 and pES31Hneo expression plasmids were constructed using pCDNA3, pCDNA3.1zeo- (Invitrogen) and the pCAGGS[45] vectors for mammalian expression. Restriction- and DNA modifying enzymes and Vent-DNA polymerase (New England Biolabs, Beverly, MA) were used as recommended by the manufacturers. Gene assembly was conducted by introduction of suitable restriction sites using modifying PCR primers. All PCR-derived fragments were sequence verified after cloning. The XhoI/BsaI fragment of pCAGGS carrying the βactin-βglobulin promoter and CMV enhancer was fused to the EcoRV/BsaI fragment of pCDNA3.1-carrying the zeocin resistance gene. An XhoI/blunt adaptor sequence was inserted 3' of the promoter and 5' of the pCDNA3.1-fragment. This adaptor incorporated a kozak sequence (GCCACCATGG) and a consensus excretion signal sequence (MGWSCIIFFLVATATGVHS). An Eco47III site was created in frame 3' of the signal sequence to allow for easy insertion of genes behind the signal sequence. At the 3' end of the gene insertion point a BspeI restriction site was provided in frame with an E-tag sequence. A BamHI site is present 3' of the E-tag sequence in front of the stop codon when no tag is to be attached. The pES31Hneo vector was constructed by fusing the pES31 BspEI/BsaI fragment carrying the promoter and signal sequence with the BspEI/BsaI fragment of pCDB1E6H2sc2C11Hneo, a derivative of pCDNA3 [32]. This fragment holds a His_6 _tag sequence for C-terminal fusion to an inserted gene and is accessible for cloning via a BspEI site. A neomycin resistance gene is also present.

The pKai51 yeast expression vector originated from pGAPZalfaA (Invitrogen). The PCR reaction with NM743 and NM744 (Table 1) primers introduced an N-terminal His_6 _tag followed by a Caspase 3 protease site to allow tag removal. The GAP promoter was replaced with AOX1 cut from pPICZA (Invitrogen) with a BglII/BstBI digest. pKai51.2 was obtained through removal of the PmeI site in the middle of the pKai51 AOX1 promoter by site-directed mutagenesis. pKai61 originated from pKai51 through a PCR reaction with NM831 and KAI41 which introduced an S-tag in front of the His6-tag.

**Table 1:** Primers used in this work, in order of appearance, 5' to 3' notation

**Name**	**Sequence 5' to 3'**
NM743	CCCGTTAACATGGTGATGGTGATGATGCATATGAGCATGCCTTTTCTCGAGAGATACCCCTTC

NM744	CCCGGCTCGGACGAAGTGGATATCTAAGCTTGAGCTCTAACTAGTTAGCCTTAGACATGACTGTTCC

NM831	AAAAGGCATGCTAAGGAGACTGCTGCCGCCAAATTCGAGAGACAACACATGGACTCCCATATGCATCATCACCATCAC

KAI41	TCTAGGACTAGTGGATCCGCACAAACGAAGGTCTCA

NM265	CAGGTCCAGCTGGTGCAGTC

NM263	TATGGATCCTTATCCGGAGGGCCCTGCGGCCGCACAAGATTTGGGCTC

NM266	CTTGAAATTGTGCTGACTCAGTCTCC

NM264	TATGGATCCTTATCCGGAGGGCCCACACTCTCCCCTGTTGAAGCTC

NM302	AGCCCCGGGCAGGTCCAGCTGGTGCAGTC

NM303	TATGGATCCTTATCCGGATCGTTTGATATCCACTTTGGTC

NM938	AGGCTAACTAGTTTAGGCGCCACGTGGTTCCAG

NM937	AGGCTAACTAGTTTATCTTTTGATATCCACTTTGGTCCCAG

### Construction of the bi- and trivalent anti-MUC1 mammalian expression vectors

Heavy and light chains of the PH1 Fab were cloned into pES31. The Fd fragment was amplified by PCR using Vent polymerase with primers NM265 and NM263. This PCR fragment was BspEI cut, kinated with T4-kinase and introduced into pES31Hneo, resulting in pES31-PH1^heavy^_neo. The L chain was cloned in pES31 in a similar fashion using primers NM266 and NM264, resulting in pES31-PH1^light^_zeo. For the construction of the bi- and tribody vectors, a PH1 scFv was fused to the C-terminus of the PH1-Fd and the PH1-L chain with a flexible linker. First, the DNA fragment coding for the flexible EPSGP(G_4_S)_3 _linker [32] was fused to the 3' end of the PH1 Fd gene with a ApaI/PvuI digest. Similarly, the DVDGGSRGDGPG linker [32] was fused to the 3' end of the PH1-L chain. Next, the PH1 scFv gene was amplified using NM302 and NM303 primers. After T4 kination, the fragment was introduced after the linker of the PH1-Fd chain using a BspeI digest. In this manner, the His_6 _tag sequence was retained and fused to the C-terminus of the PH1 scFv, resulting in pES31-PH1^heavy^-scFv_neo. For cloning the PH1 scFv to the linker behind the PH1-L chain, a SmaI/BamHI fragment was used. This was fused to an Eco47III/PvuI fragment and a BamHI/PvuI fragment from pES31, yielding pES31-PH1^light^-scFv_zeo.

### Construction of antibody expressing plasmids for Pichia pastoris

Using the mammalian vectors, the PH1 light chain was amplified from pES31-PH1^light^_zeo with NM266 and NM938 and cloned into pKai51.2 with an EcoRV/SpeI cut, resulting in pKai51.2-PH1^light^. The heavy chain PH1 was amplified from pES31PH1^heavy^_zeo with NM265 and NM938 and cloned into pKai61 with a EcoRV/SpeI cut, resulting in pKai61-PH1^heavy^. pKai51.2-PH1^light^-scFv, containing the light chain PH1-scFv PH1, was made by cloning the EcoRV/SpeI digested amplification fragment from pES31-PH1^light^-scFv_zeo with primers NM266 and NM937. Similarly, the PH1 heavy chain-scFv PH was amplified from pES31-PH1^heavy^-scFv_neo with NM265 and NM937, cut with EcoRV/SpeI and cloned in pKai61, resulting in pKai61-PH1^heavy^-scFv. Finally, vectors containing 2 expression modules for the heavy and light chain were assembled as follows: pKai61-BiBody-PH1 is the ligation product of pKai61-PH1^heavy^-scFv cut with BamHI/BsrGI and pKai51.2-PH1^light ^cut with BglII/BsrGI. pKai61-TriBody-PH1 is a combination of pKai61-PH1^heavy^-scFv cut with BamHI/BsrGI and pKai51.2-PH1^light^-scFv cut with BglII/BsrGI.

### Transient Expression in HEK293T cells

For transient antibody expression, HEK293T cells were transfected according to the Ca_3_(PO_4_)_2 _precipitation method [46]. In brief, cells were seeded at 4 × 10^6 ^cells/175 cm^2^, 20 h before transfection, after which 14 μg DNA of each expression plasmid was added to the cells for 24 h. For expression of the bibody pES31-PH1^heavy^-scFv_neo and pES31-PH1^light^_zeo were used. The tribody was expressed by combining the pES31-PH1^heavy^-scFv_neo and pES31-PH1^light^-scFv_zeo plasmids. The cells were then covered with supplemented DMEM containing 5 mg/l bovine insulin, 5 mg/l transferrin and 5 μg/l selenium (ITS). Medium was harvested every 48 h. For both the bi- and tribody production, 1600 ml HEK293T supernatant was collected.

### Stable expression in NS0 cells

NS0 cells were transfected by electroporation with pES31-PH1^heavy^-scFv_neo and pES31-PH1^light^_zeo for the bibody, pES31-PH1^heavy^-scFv_neo and pES31-PH1^light^-scFv_zeo for the tribody. 2.5 μg of each plasmid was used with Nucleofector technology (Amaxa Biosystems, Cologne, Germany) and program settings G16 and T27 in buffer T as indicated by the manufacturer. Prior to transfection, DNA was sterilised on a 0.22 μm filter. Linearised DNA was obtained through a ScaI digest, followed by a Wizard DNA cleanup procedure (Promega, Leiden, NL). Control transfections were performed using 5 μg pmaxGFP plasmid. After 24 h–48 h transfected cells were evaluated for GFP expression and put on selective medium containing 0.6 mg/ml G418 (Invitrogen) and/or 0.6 mg/ml zeocin (Invitrogen). Mock transfections were performed using H_2_O instead of DNA. After 1 month of selection, subclones were generated using limiting dilution. ELISAs were performed to ascertain bi- and tribody clone production levels. The best bibody and tribody clone were adapted to protein free hybridoma medium and grown in roller bottles for 7 days. For each clone 3 roller bottles of 670 ml NS0 supernatant/bottle were collected.

### Stable expression in P. pastoris yeast cells

Prior to transformation 10 μg of the bicistronic expression vectors pKai61-BiBody-PH1 and pKai61-BiBody-PH1 were linearised with a PmeI digest. The plasmid DNA (2.5 μg) was transformed into 100 μl competent P. pastoris GS115 cells by electroporation using a Gene Pulser (Bio-Rad) and 0,2 cm gap cooled electroporation cuvettes. The electric pulse parameters were: 1500 V, 40 μF, 200 Ω and 8 ms duration. Immediately after the pulse, 1 ml of 1 M ice-cold sorbitol was added. This mixture was transferred to tubes containing 2 ml YPD medium. These tubes were incubated at 30°C for 1 – 1.5 hour without shaking. 50 μl was subsequently plated on YPD-agar plates containing 100 μg/ml zeocin and incubated at 28°C for 3–4 days. Colonies were screened for expression through small-scale expression experiments in 24 well deep-well plates containing 2 ml YPNM medium. After 48 h of methanol induction, several clones were compared by means of an S-Tag assay. Production of the bibody and tribody was performed in shake flasks containing 250 ml YPNM medium/flask.

### Purification

The HEK293T derived bi- and tribodies were purified using an adaptation of a previously described protocol for mouse bi- and tribodies [32]. Harvested mammalian medium was centrifuged for 20 min at 13000 × g and filtered over 0.22 μm bottle-top filters (Nalgene, Neerijse, BE). Having a predicted pI of 7.9 and 8.0 respectively, the pH of the bi -and tribody HEK293T samples was adjusted to pH 5.5 with acetic acid before loading on a 200 ml XK50/20 SP Sepharose Fast Flow (GE Health, Uppsala, Sweden) cation exchange chromatography (CEC) column equilibrated with 50 mM NaAc buffer pH 5.5 and run at 9 ml/min. Further purification of the CEC fractions was performed using 1 ml Hitrap columns (GE Health) loaded with Ni^2+^. The sample was supplemented with 20 mM Imidazole pH 7.5 and pH was adjusted to 7.5. The column was equilibrated using 20 mM phosphate (PP) buffers containing 0.5 M NaCl and run at 2 ml/min. For elution 20 mM PP buffer with 200 mM or 400 mM imidazole and 0.5 M NaCl were used. IMAC of serum free NS0 samples was performed on a 20 ml C16/20 Chelating Sepharose Fast Flow column (GE Health), under the same buffer conditions as the 1 ml Hitrap columns, but using a gradient elution with 20 mM PP buffer containing 500 mM imidazole and 0.5 M NaCl. For size exclusion chromatography (SEC), the sample was concentrated to 2 ml using Centricon centrifugal filter devices (Amicon Bioseperations, Beverly, MA), with a cut-off of 10 kDa. Size exclusion chromatography was performed on a 120 ml C16/60 Sephacryl S-200 high resolution grade column (GE Health) and run at 0.5 ml/min with PBS. The column was calibrated before each run with Gel Filtration Standard (Bio-Rad Laboratories, Hercules, CA).

Harvested yeast medium was precipitated by addition of 70% (NH_4_)_2_SO_4_. After centrifugation, the pellet was dissolved in 10 ml 20 mM NaH_2_PO_4_, 300 mM NaCl, 20 mM imidazole pH 7.5. This solution was injected on a 62 ml desalting sephadex G-25 column (XK16/31) with the same buffer. The desalted protein fraction was further purified on a 6 ml Ni sepharose column for the bibody and on a 1 ml HisTrap column for the tribody. The column was equilibrated with 20 mM NaH_2_PO_4_, 300 mM NaCl, 20 mM imidazole pH 7.5, washed with 50 mM imidazole and eluted by 400 mM imidazole in the same buffer. The eluted protein fraction was finally purified by 1 ml injections on a Superdex 200 HR10/30 column to PBS. All liquid chromatography (LC) runs were performed at 4°C on Pharmacia FPLC or Akta purifier systems (GE Health).

### Analysis of protein fractions

Collected protein fractions were analysed by SDS-PAGE, after precipitation with TCA. Proteins were visualized using Coomassie Brilliant Blue dye or Western blotted to a nitrocellulose membrane. Immunodetection of the proteins on the blot was performed by incubating consecutively with mouse IgG1 anti-human κ serum, mouse IgG1 anti-human Fd serum (Sigma Aldrich, St. Louis, MO) or mouse IgG1 anti-His (Qiagen, Venlo, NL) and alkaline phosphatase (AP) conjugated anti-mouse IgG1 (Becton Dickinson-Pharmingen, Erembodegem, BE). Alternatively, for yeast constructs S-protein-AP (Merck, Nothingham, UK) was used to detect western blots. Antibodies were incubated for 1 h at room temperature (RT) in a solution containing 5% skimmed milk powder, 50 mM Tris pH 8.0, 0.47% NaCl, 0.2% NP40 and 0.02% NaN_3_. Subsequent visualisation was performed with NBT/BCIP substrate 'ready to use tablets' (Roche, Vilvoorde, BE). Protein recovery and purity were determined on Coomassie gels that were scanned and analysed with Quantity One software (Bio-rad). Protein concentrations of pure protein were measured with the Micro BCA™ Protein Assay Reagent Kit (Pierce, Rockford, IL) with IgG standard protein. Concentrations of PH1 molecules from *P. pastoris *medium were quantified with the FRETWorks S-tag assay kit (Merck) according to the instructions of the manufacturer. Yields were calculated as: (amount of bi- or tribody withheld in elution)/(total amount of bi- or tribody found before the purification step). Additionally for gelfiltrations, the purity was calculated by measuring the area under the curve for the total run compared to the specific bi- or tribody peak. The overall yield was calculated by dividing the ultimate quantity of pure bi/tribody by the estimated starting quantity before purification.

### ELISA

96 well plates were coated overnight at RT with 50 μl mGroEL-MUC1 (10 μg/ml) in NaHCO_3 _buffer pH 9.6. Plates were blocked with PBS buffer + 0.05% Tween20 and 1% BSA (PBS-BSA) for 2 h at 37°C. Dilution series of the Ab-derivatives were added in 50 μl PBS-BSA and incubated for 1 h at 37°C. In between steps, plates were washed 3 times with PBS + 0.05% Tween20. Detection was performed by incubating consecutively with mouse IgG1 anti-human κ serum and AP-conjugated anti-mouse IgG1. After adding the p-nitrophenyl phosphate (PNPP) substrate in 10% diethanolamine + 1 mM MgCl_2 _pH 9.8, absorbance at 405 nm was measured in a microplate reader (Bio-Rad). Negative controls consisted of wells treated with antibodies but not coated with antigen and wells that were coated and received detection antibodies, but no samples. ELISA binding curves and K_D _were calculated using Prism 4.0 software (Graphpad, San Diego, CA).

### Flow cytometry

Aliquots of 2 × 10^5 ^cells were incubated with PH1 derivatives in PBS + 0.5% BSA and 0.02% NaN_3 _(PBS-A) on ice for 2 h. After washing, the cells were allowed to subsequently interact with mouse IgG1 anti-human κ and Alexa Fluor 488-conjugated goat anti-mouse antibodies (Invitrogen) on ice for 1 h. Finally, the cells were resuspended in 300 μL PBS-A. For isotype control, 10 nM 9E10 MoAb (mouse IgG1, Becton Dickinson-Pharmingen) was used instead of PH1 derivatives. Flow cytometric analysis was performed using a FACScan flow cytometer (Becton Dickinson). Data analysis based on the collection of 10.000 events per sample was performed using WinMDI 3.7 software (Joseph Trotter).

## Authors' contributions

SS conceived the bivalent and trivalent anti-MUC1 molecules based on the Fab-scFv model thought out by NM. The pES vector system was designed by NM and used by SS to construct all mammalian expression plasmids. Expression and purification of mammalian produced bi- and tribodies was performed by SS, as well as ELISA and flow cytometric characterisations. VK and MZ designed and produced the stable *Pichia pastoris *clones expressing the PH1 bi -and tribodies. CD and JH produced and purified the PH1 bi-and tribodies from these *Pichia *clones and performed initial quality checks of the products. Finally, JG participated in the coordination of the study and had a major role in drafting the manuscript together with SS. All authors read and approved the final version of the manuscript.
